# Thyroid Hormone Role on Cerebellar Development and Maintenance: A Perspective Based on Transgenic Mouse Models

**DOI:** 10.3389/fendo.2014.00075

**Published:** 2014-05-20

**Authors:** Larissa C. Faustino, Tania M. Ortiga-Carvalho

**Affiliations:** ^1^Laboratorio de Endocrinologia Molecular, Instituto de Biofisica Carlos Chagas Filho, Universidade Federal do Rio de Janeiro, Rio de Janeiro, Brazil

**Keywords:** thyroid hormones, genes, cerebellum, brain development, animal models

## Abstract

Cerebellum development is sensitive to thyroid hormone (TH) levels, as THs regulate neuronal migration, differentiation, and myelination. Most effects of THs are mediated by the thyroid hormone receptor (TR) isoforms TRβ1, TRβ2, and TRα1. Studies aimed at identifying TH target genes during cerebellum development have only achieved partial success, as some of these genes do not possess classical TH-responsive elements, and those that do are likely to be temporally and spatially regulated by THs. THs may also affect neurodevelopment by regulating transcription factors that control particular groups of genes. Furthermore, TH action can also be affected by TH transport, which is mediated mainly by monocarboxylate transporter family members. Studies involving transgenic animal models and genome-wide expression analyses have helped to address the unanswered questions regarding the role of TH in cerebellar development. Recently, a growing body of evidence has begun to clarify the molecular, cellular, and functional aspects of THs in the developing cerebellum. This review describes the current findings concerning the effects of THs on cerebellar development and maintenance as well as advances in the genetic animal models used in this field.

## Introduction

The thyroid hormones (THs) thyroxine (T_4_) and 3,5,3′-triiodothyronine (T_3_) are essential for embryonic development and play critical roles in cellular metabolism, acting primarily through the stimulation of oxygen consumption and basal metabolic rate (1, 2). THs are necessary for proper central nervous system (CNS) development, and they have long been known to regulate neuronal differentiation and migration, synaptogenesis, and myelination (3–6). The cerebellum is located near the rear of the brain stem at the midbrain–hindbrain junction, and this structure is generally thought to coordinate proprioceptive–motor functions, although more recently, it has also been associated with neurocognition (7, 8). The cerebellum was one of the first targets of THs to be identified, and it is a useful model for studying the mechanisms by which THs influence the CNS. In particular, the cerebellum has a relatively homogenous and simple structure with a well-characterized laminar organization and a small number of cell types that develop within spatially defined regions (9–11).

The majority of TH actions are mediated through the binding of T_3_ to nuclear thyroid hormone receptors (TRs), which act as ligand-modulated transcription factors that modify the expression of target genes (12). Fundamentally, TH nuclear signaling is mediated by interactions between TRs and specific DNA sequences known as thyroid response elements (TREs), which associate with a variety of co-factors within the regulatory regions of target genes (12, 13). TR isoforms are expressed in several brain regions, including the cerebellum (14, 15). However, the target genes of THs and the cells that express genes likely to be involved in cerebellar development and maintenance are still not well-established (6, 16).

In addition to the classical roles of TH in the nucleus, TH can also initiate rapid effects at the cell surface, within mitochondria and via cytoplasmic TRs (17, 18). The fact that brain development in TR knockout (KO) animals is only slightly affected (19) suggests the existence of non-genomic morphogenic roles for TH in the CNS. One of the best characterized non-genomic roles for TH in the brain is illustrated by the induction of actin polymerization in astrocytes by T_4_ *in vitro* (20), which is very important for the organization of extracellular neural guidance molecules during neurodevelopmental processes. Finally, TH metabolism and transport, which are mediated mainly by deiodinases (21) and monocarboxylate transporters (22, 23), respectively, have also been shown to be important for cerebellar function.

The aims of this review are to briefly describe the current knowledge concerning the effects of THs on cerebellar development and functional maintenance as well to summarize advances in the genetic animal models used in this field.

## The Influence of THs on Cerebellar Ontogenesis

In humans, T_3_, T_4_, and TRs are already present within the developing cortex prior to the onset of fetal thyroid gland activity, or gestational week 12, which suggests an important role for maternal TH during this critical window of brain development (24–27). Congenital hypothyroidism leads to structural and intellectual impairment in infants (28). Furthermore, TH administration to human infants with congenital hypothyroidism immediately after birth was shown to promote near-normal intellectual development (29). The majority of studies on the role of THs in neurodevelopment have been carried out in rodent models in which THs, deiodinases, and TRs are present prior to the onset of fetal TH synthesis and secretion (30, 31). *Paired box 8* (*Pax8*) KO mice are a commonly used animal model for studying the effects of postnatal TH on CNS development, as Pax8 is an essential transcription factor for thyroid follicular cell differentiation, and its absence leads to thyroid gland dysgenesis (32). Therefore, the *Pax8*-KO mouse is a model for congenital hypothyroidism that displays extensive abnormalities in cerebellar development, resulting in an ataxic phenotype (32–34) (Table 1).

**Table 1:** **Summary of mutant animal models and their cerebellar phenotypes**.

Animal model	Etiology	HPT axis	Brain TH state	Cerebellar phenotype	Locomotor behavior	Reference
*Pax8*-KO	Pax8 knockout	Thyroid gland dysgenesis	Increased TRH and TSH expression; elevated cerebellar D2 activity; decreased cerebellar D3 activity	Increased cell number in the EGL; reduced dendritic growth in Purkinje cells	Ataxic phenotype	(32, 33, 35)
*Slc16a2* KO	MCT8 knockout	Elevated serum levels of T_3_ and TSH; decreased serum levels of T_4_	Reduced T_3_ and T_4_ brain content; increased TRH expression; increased cerebellar D2 activity; decreased cerebellar D3 activity	Milder neurological phenotype than that observed in patients; no alterations in Purkinje cells	Locomotor activity similar to WT mice	(35 –38)
*Pax8/Slc16a2* double KO	Pax8 and MCT8 knockout	Thyroid gland dysgenesis	Increased TRH and TSH expression; increased cerebellar D2 activity; decreased cerebellar D3 activity	Reduced dendritic arborization; thinner molecular layer		(35)
*Slco1c1* KO	OATP1C1 knockout	Normal serum T_3_ and T_4_ levels	Mild decrease in T_4_ brain content; normal T_3_ brain content	Normal Purkinje cell morphology	Normal motor activity on rotarod test	(39)
*Slco1c1/Slc16a2* double KO	OATP1C1 and MCT8 knockout	Elevated serum levels of T_3_ and TSH; decreased serum levels of T_4_	Brain-specific hypothyroidism increased TRH expression; elevated cerebellar D2 activity; reduced cerebellar D3 activity	Impaired arborization and dendritic growth of Purkinje cells at P12; no alterations in Purkinje cells at P33 or P120	Impaired motor coordination and locomotor activity	(38)
*Slc7a8* KO	LAT2 knockout	Normal serum T_3_, T_4_, and TSH levels	Normal TSH expression; normal pituitary D2 expression; normal cerebellar D3 expression	Normal cerebellar gene expression and morphology	Mildly impaired movement coordination on rotarod test	(40)
*Dio2*-KO	D2 knockout	Normal serum T_3_ levels; elevated serum T_4_, and TSH levels	Decreased T_3_ brain content; increased brain D3 activity	Milder alterations in cerebellar TH-responsive genes (*Srg1* and *Hr*) than in hypothyroidism		(41, 42)
*Dio3*-KO	D3 knockout	Increased serum T_3_ levels during perinatal development	Brain thyrotoxicosis; increased cerebellar D2 activity; reduced cerebellar D3 activity	Upregulated cerebellar TH-responsive genes (*Hr*); impaired cerebellar foliation; early dissipation of EGL; rapid expansion of the molecular layer	Defective locomotor activity on vertical pole and rotarod test	(43 –45)
*Thra*^−/−^	TRα1 deletion	Normal serum T_3_ levels; slightly decreased serum T_4_ levels; reduced serum TSH levels	Decreased TSHα expression; increased TSHβ expression	Non-hypothyroid cerebellar phenotype	Normal locomotor activity	(46, 47)
*Thrb*^−/−^	All TRβ deletion	Increased levels of TSH, T_3_, and T_4_	Increased T_3_ brain content decreased TSH expression	No alterations in TH-responsive genes in the cerebellum	No behavioral defects	(48, 49)
*Thrb* Δ337T	TRβ mutation	Elevated levels of T_3_, T_4_, and TSH	Hypothyroid-like brain (low levels of TH-responsive genes BDNF and *Pcp2*)	Impaired cerebellar foliation; altered laminar organization; abnormal Purkinje cell dendritogenesis; reduced Bergmann glia fibers; reduced cerebellar gene expression (*Pcp2*)	Severe impairment in balance and coordination	(50, 51)
*Thra* PV	TRα1 mutation	Mild increase of T_3_, T_4_, and TSH levels		Reduced cerebellar gene expression (*Srg1*)		(52)
*Thra* R384C	TRα1 mutation	Normal serum levels of T_4_, T_3_	Normal TSH expression	Delayed migration of EGL to IGL; mild alterations of Purkinje cells	Reduced locomotor activity	(53, 54)
*Thra* L400R	TRα1 mutation	Normal serum levels of T_4_, T_3_	Normal TSH expression Hypothyroid-like brain (low levels of TH-responsive genes)	Late granule cell differentiation pattern similar to congenital hypothyroidism; mild alterations of Purkinje cell arborization; low expression of TH-responsive genes (*Hr* and *Pcp2*); delayed loss of Purkinje cells axonal regenerative capacity; impaired differentiation of Purkinje cells and Bergmann glia		(55 –58)
*Ncoa1*^−/−^	SRC-1 deletion	Elevated TSH, T_4_, and T_3_ levels		Delayed Purkinje cells development and maturation	Reduced motor coordination and strength	(59)

*BDNF, brain-derived neurotrophic factor; EGL, external granular layer; IGL, internal granular layer; *Srg1*, synaptotagmin-related gene 1, *Hr*, hairless; *Pcp2*, Purkinje cell protein 2*.

Rodent cerebellar development is complete within the first 2–3 weeks after birth, when the cerebellar foliation process, which encompasses the transition from a smooth cerebellar surface to an X lobule cerebellum, is completed (7). It has long been known that cerebellar ontogenesis is closely linked to TH regulation (60–62), although the molecular mechanisms through which THs modulate this process remain unclear. Hypothyroidism results in a number of morphological alterations in the cerebellum, including increased neuronal death within the internal granular layer (IGL), increased perdurance of the external granular layer (EGL), defects in granular cell migration, impaired Purkinje cell dendritogenesis, delayed myelination, defects in the late differentiation pattern of Golgi interneurons and mossy fibers, reduced protrusions of Bergmann glial cells, and increased cell apoptosis (9, 46, 63–65). TH administration prior to the end of postnatal week 2 prevented these structural changes. Moreover, the expression levels of neurotrophins and growth factors, such as BDNF, NT3, and EGF, as well as cell adhesion molecules, such as NCAM and L1, are modified by TH in the developing cerebellum (63, 66–68). For example, TH was shown to promote cerebellar neuronal migration and the differentiation of Bergmann glia by inducing EGF secretion (69).

## Perspectives from Transgenic Mouse Models

T_3_ and T_4_ enter the cell through plasma membrane transporters, including the monocarboxylate transporter family members MCT8 and MCT10, organic anion transporting peptides (OATP), and carriers of l-amino acids (LATS) (70, 71). Recent studies have indicated that TH transporters such as MCT8, which are found in a subset of neuronal populations (23), may play critical roles in neurodevelopment processes mediated by THs. Patients harboring inactivating mutations in the MCT8 gene (*Slc16a2*) exhibit Allan–Herndon–Dudley syndrome, which is characterized by psychomotor retardation, lack of speech development, increased serum T_3_ concentrations, and low T_4_ levels (72, 73).

Although MCT8-KO mice have been generated, they do not display the same neurological abnormalities observed in human patients (Table 1). This phenomenon is likely due to the presence of other neuronal TH transporters, such as OATP14, LAT1, and LAT2, during earlier stages of mouse brain development that compensate for the absence of MCT8 (36, 74). However, another possible explanation for the difference between the mouse and human phenotypes is that human MCT8 is necessary for the transport of an unknown signaling molecule necessary for CNS development, which is consistent with clinical evidence indicating that the neurological syndromes observed in patients with MCT8 mutations are more severe than those observed in patients with congenital hypothyroidism (36). A recent study performed in MCT8-KO mice demonstrated that 3,5,3′,5′-tetraiodothyroacetic acid (tetrac), a T_4_ metabolite that is not transported by MCT8 or OATP1C1, is capable of replacing TH during brain development (35). Tetrac can be converted into 3,3′,5-triiodothyroacetic acid (triac) by deiodinase type 2, which can subsequently interact with TRs, thereby replacing T_3_ activity. Indeed, treatment of MCT8-KO mice with tetrac led to improvements in TH-dependent neuronal differentiation in the striatum, cortex, and cerebellum during the first three postnatal weeks.

A mouse model lacking LAT2 (*Slc7a8*) was generated to further characterize the role of this transporter in TH physiology. However, LAT2-KO mice exhibited normal cerebral and cerebellar development, with the exception of slight defects in movement coordination on rotarod tests (40) (Table 1).

The iodothyronine deiodinase enzymes D1 (*Dio1*) and D2 (*Dio2*) modulate the intracellular availability of the active hormone T_3_. In particular, D2 catalyzes the conversion of T_4_ to T_3_, whereas D3 inactivates T_4_ and T_3_ by converting them to T_2_ and reverse T_3_ (rT_3_), respectively (75). Studies have demonstrated that nearly 80% of T_3_ is generated by local conversion within the brain (3, 5) through the activity of D2, which is primarily found in astrocytes (41). Therefore, the presence of D2 together with increased levels of T_3_ suggests a role for D2 in supplying the developing brain with T_3_ derived from maternal T_4_. However, some unexpected findings in *Dio2*-KO mice are inconsistent with the hypothesis that D2 is essential for all TH-dependent neurodevelopment processes.

Although *Dio2*-KO mice display elevated brain T_4_ levels and reduced T_3_ content, surprisingly, the observed neurological impairments, which included changes in the cerebellar expression of TH-dependent genes and behavioral defects, were found to be mild compared with those observed in hypothyroidism (42, 76). These data suggest that decreased local T_3_ production can be largely compensated for by increased T_3_ uptake from circulation, and indeed, this was later confirmed by experiments carried out in double *Dio1*/*Dio2*-KO mice, which demonstrated normal serum T_3_ concentrations and only mild neurological phenotypes (21). On the other hand, *Dio3*-KO animals were characterized by high T_3_ levels during perinatal development, which induced the upregulation of TH-responsive genes in the cerebellum (43, 44). Recently, it was reported that *Dio3*-KO mice exhibited impaired cerebellar foliation, early premature disappearance of the EGL, rapid expansion of the molecular layer, and abnormal locomotor behavior. Furthermore, the cerebellar phenotypes of these mice could be partially rescued by deletion of the TRα1 isoform (45) (Table 1).

**Figure 1:**
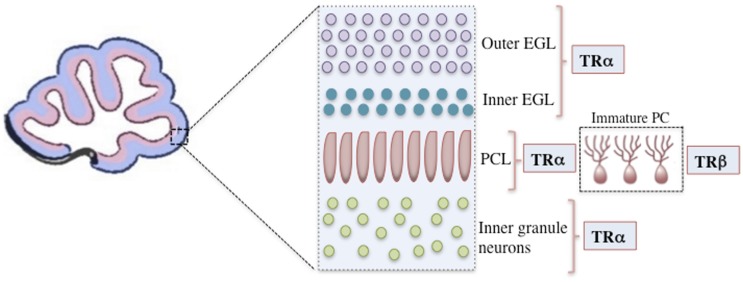
**A representation of the mouse cerebellar cortex during the initial postnatal days showing the positions of cells expressing specific TR isoforms**. Only the outer EGL, inner EGL, Purkinje cell layer (PCL), and inner granule layer are shown. TRα is primarily expressed in granular cell precursors and subsequently in the transient outer and inner EGL. In the Purkinje cells, TRα is the first isoform to be detected, however, after the second postnatal week TRβ is predominantly expressed. TR, thyroid hormone receptor; EGL, external granular layer; P14, postnatal day 14; PCL, Purkinje cell layer.

The majority of TH functions are mediated through nuclear TRs, which are members of a superfamily of ligand-modulated transcription factors that can either upregulate or downregulate target gene transcription (2). The consensus for positively regulated genes is that TRs bind to activating TREs both in the presence and absence of T_3_. In the absence of T_3_, TR represses target gene transcription by recruiting co-repressors, whereas in the presence of T_3_, co-repressors are released and co-activators are recruited, leading to transcriptional up regulation (1, 12). In mammals, two different genes encode at least three high-affinity TRs: TR-β1 (*Thrb*), TR-β2 (*Thrb*), and TR-α1 (*Thra*) (77). TR-α1 is the isoform that is predominantly expressed both prenatally and postnatally throughout the brain, including the developing cerebellum, and it is responsible for nearly 80% of total receptor T_3_ binding (14, 78, 79). In contrast, TR-β expression is confined to a few postnatal neuronal populations, including the paraventricular hypothalamus, cerebellar Purkinje cells, and hippocampal pyramidal and granule cells (80, 81). In rodents, TR-α1 is already present at E11.5 in the neural tube and at E12.5 in the diencephalon and ventral rhombencephalon (14). Both TRα and TRβ are expressed in the cerebellum. TRα is primarily expressed in the early cerebellar neurepithelium, granular cell precursors, and later in the transient EGL, whereas TRβ is predominantly expressed during later stages, notably in the Purkinje cell layer (PCL) and in deep internal layers (14, 81, 82) (Figure 1).

*Thra*- and *Thrb*-KO mouse models, which exhibit abrogated nuclear signaling, have been created to address the roles of different TR isoforms in proper brain development and function (47, 48, 83). However, it was reported that these mice exhibit only a mild neurological phenotype compared with hypothyroid animals, indicating that the absence of T_3_ binding (unliganded TR) is more harmful to the CNS than the absence of TR isoforms (46, 84) (Table 1). Later, *Thra*- and *Thrb*-knock-in mutant mice expressing dominant-negative TRs were generated, and it was reported that these mice were phenotypically distinct from TR-KO mice (50, 53–55). Specifically, in mice harboring the *Thrb* Δ337T mutation – a point mutation in the ligand-binding domain that prevents T_3_ binding but not binding to DNA or co-factors (85) – cerebellar morphogenesis was similar to that observed in congenital hypothyroidism, presumably because TR remained constitutively bound to its co-repressors, thereby mimicking a hypothyroid state (50). Hashimoto et al. (50) demonstrated that *Thrb* Δ337T mice displayed impairments in balance and coordination, reductions in the molecular and PCLs, and decreases in the number and branching of Purkinje cells, which may account for the decreased cerebellar size observed in these mutant animals.

Therefore, functional TR-β is required for TH-dependent cerebellar development, which was further demonstrated by the phenotypes observed in *Thrb* Δ337T mutant mice, including defects in cerebellar foliation, altered laminar organization, abnormal Purkinje cell dendritogenesis, and reduced Bergmann glia fibers (51). Cerebellar foliation is characterized by the presence of 10 well-formed lobules and sub-lobules (7). In *Thrb* Δ337T homozygotes at postnatal day (PND) 21, researchers observed decreases in the molecular and granular layers as well as a failure in the subdivision of lobule VI, which is subdivided into sub-lobules VIa and VIb in wild-type and heterozygous animals. During PND 9, which is the initial period of cerebellar development, *Thrb* Δ337T mice fail to form fissures between lobules VI–VII, and lobule IX is also severely affected. During both the initial and final stages of cerebellar foliation, the *Thrb* Δ337T mutation leads to extreme defects in fissure and lobule formation (51). Unfortunately, the identification of direct target genes that are regulated by TH in the developing brain using RNA-based techniques has been problematic. However, recent studies using chromatin immunoprecipitation combined with DNA microarray analysis (ChIP on chip) identified a large number of TR-β binding sites and target genes in the developing mouse cerebellum, reinforcing the role of TR-β in mediating gene transcription through TH in this brain structure (86, 87). Chatonnet et al. introduced TR-α1 and TR-β1 into a neural cell line lacking endogenous TRs and demonstrated that the majority of the T_3_ target genes analyzed were regulated by both TR-α1 and TR-β1. Nevertheless, a significant number of the analyzed genes showed strong preferences for one receptor isoform over the other (88).

In the cerebellum of mice carrying a cell-specific L400R mutation in the ligand-binding domain of TR-α1 *Thra* L400R), which prevents histone acetyltransferase recruitment and facilitates the permanent recruitment of co-repressors, there is a delay in the pattern of granule cell differentiation similar to what is observed in congenital hypothyroid animals; however, Purkinje cell arborization is not strongly affected in these mutants (55). Another study involving *Thra* L400R mice highlighted the importance of TRα-dependent signaling in postnatal brain development by showing that it promotes the secretion of neurotrophins from astrocytes and Purkinje cells and that it maintains adult brain function by limiting the proliferation of oligodendrocyte precursor cells (56). Late in their development, these mutant mice displayed a loss of axonal regenerative capacity in Purkinje cells, which is thought to play a role in the brain maturation process. These data indicate an important role for TR-α1 in mediating T_3_-induced inhibition of axonal regeneration in Purkinje cells (57). In addition, it was very recently reported that the L400R mutation primarily affects the differentiation of two specific cerebellar cell populations, Purkinje cells, and Bergmann glia, which indicates that the autonomous effects of TH on these cells indirectly impact global cerebellar cortex development (58). In Purkinje cells, T_3_ acts through TR-α1 to promote dendritic tree development and the secretion of neurotrophic factors, whereas in Bergmann glia, T_3_ promotes the development and organization of radial fibers and the alignment of cell bodies within the PCL (58) (Table 1). In humans, a role for TR-α1 in brain development is supported by descriptions of patients with cognitive impairment phenotypes similar to those observed in congenital hypothyroidism who harbor primary mutations in the *THRA* gene (89, 90).

Taken together, these data suggest that TR-α and TR-β function together to mediate the processes of cerebellar ontogenesis controlled by THs. Compared with *Thrb* mutants, *Thra*-knock-in mice show more severe cerebellar defects, indicating that TR-α may play a key role in regulating the expression of target genes involved in cerebellar ontogeny (52). Other relevant mutant animal models with impaired neurological phenotypes also exist, such as *Ncoa1*-KO animals. Steroid receptor co-activator 1, which is encoded by the *Ncoa1* gene, has been shown to modulate TH activity via specific TR isoforms (91, 92). This co-activator is highly expressed in the cerebellum; thus, *Ncoa1*-KO mice exhibit cerebellar abnormalities that are similar to those observed in congenital hypothyroid mice (59).

## Concluding Remarks

It has been known for decades that cerebellar development is regulated by THs. Although the molecular mechanisms through which THs impact CNS development are becoming better understood, primarily due to studies in genetic animal models, many issues remain to be addressed. Only a few T_3_ targets in neural cells have been described to date, it is important to identify additional direct target genes of THs and to determine how these genes are temporally and spatially regulated during specific neurodevelopment. Finally, the rapid non-genomic actions of THs and the role of the recently described thyronine derivatives require further analysis. Therefore, additional studies will be necessary before our model of TH activity within the developing cerebellum is complete.

## Conflict of Interest Statement

The authors declare that the research was conducted in the absence of any commercial or financial relationships that could be construed as a potential conflict of interest.
